# Characterization of the Impact of Density Gradient Centrifugation on the Profile of the Pig Sperm Transcriptome by RNA-Seq

**DOI:** 10.3389/fvets.2021.668158

**Published:** 2021-07-19

**Authors:** Yu Lian, Marta Gòdia, Anna Castello, Joan Enric Rodriguez-Gil, Sam Balasch, Armand Sanchez, Alex Clop

**Affiliations:** ^1^Centre for Research in Agricultural Genomics (CRAG), CSIC-IRTA-UAB-UB, Campus UAB, Barcelona, Spain; ^2^Unit of Animal Science, Department of Animal and Food Science, Autonomous University of Barcelona, Barcelona, Spain; ^3^Unit of Animal Reproduction, Department of Animal Medicine and Surgery, Autonomous University of Barcelona, Barcelona, Spain; ^4^Grup Gepork S.A., Barcelona, Spain; ^5^Consejo Superior de Investigaciones Científicas, Barcelona, Spain

**Keywords:** sperm RNA, RNA-Seq, sperm purification, differentially abundant gene, somatic cell, germline cell, exosome

## Abstract

RNA-Seq data from human semen suggests that the study of the sperm transcriptome requires the previous elimination from the ejaculates of somatic cells carrying a larger load of RNA. Semen purification is also carried to study the sperm transcriptome in other species including swine and it is often done by density gradient centrifugation to obtain viable spermatozoa from fresh ejaculates or artificial insemination doses, thereby limiting the throughput and remoteness of the samples that can be processed in one study. The aim of this work was to evaluate the impact of purification with density gradient centrifugation by BoviPure^TM^ on porcine sperm. Four boar ejaculates were purified with BoviPure^TM^ and their transcriptome sequenced by RNA-Seq was compared with the RNA-Seq profiles of their paired non-purified sample. Seven thousand five hundred and nineteen protein coding genes were identified. Correlation, cluster, and principal component analysis indicated high—although not complete—similarity between the purified and the paired non-purified ejaculates. 372 genes displayed differentially abundant RNA levels between treatments. Most of these genes had lower abundances after purification and were mostly related to translation, transcription and metabolic processes. We detected a significant change in the proportion of genes of epididymal origin within the differentially abundant genes (1.3%) when compared with the catalog of unaltered genes (0.2%). In contrast, the proportion of testis-specific genes was higher in the group of unaltered genes (4%) when compared to the list of differentially abundant genes (0%). No proportion differences were identified for prostate, white blood, lymph node, tonsil, duodenum, skeletal muscle, liver, and mammary gland. Altogether, these results suggest that the purification impacts on the RNA levels of a small number of genes which are most likely caused by the removal of epididymal epithelial cells but also premature germinal cells, immature or abnormal spermatozoa or seminal exosomes with a distinct load of RNAs.

## Introduction

Despite being a matter of debate for many years, the presence and role of sperm RNA is beginning to be elucidated. The analysis of the spermatozoon transcriptome can shed light on the previous, present or future roles of the genes involved in spermatogenesis, early embryo development, and transgenerational inheritance (1). The sperm's transcriptomic landscape has been profiled in a large number of animal species including human, mice, cattle, pig, and horse (1), and several research groups focus their efforts to identify molecular markers associated to semen quality and fertility in human (2), cattle (3), horse (4) and swine (5–10). Transcriptomic evaluations of the ejaculated spermatozoa are often preceded by a purification step to remove somatic and prokaryotic cells often by using a commercial colloidal silica suspension (e.g., PureSperm®, BoviPure^TM^, Nidacon, Sweden) in order to prepare density gradients and purify the sperm cells (11–14). This step is considered to be necessary since spermatozoa carry small amounts of RNA that are also highly fragmented when compared with the somatic cells that could be present in the ejaculate. The sperm transcriptome profile could be thus overshadowed by the profiles from these somatic cells. However, the purification step with gradient cell centrifugation requires viable spermatozoa only available in fresh ejaculates or in frozen straws prepared for artificial insemination. The use of fresh ejaculates limits the number of samples that can be simultaneously processed in an experiment and the remoteness of their geographical location. In terms of logistics, studies requiring large number of samples would benefit if they could extract RNA from frozen material. In pigs, ejaculates from artificial insemination studs often contain small amounts of epithelial cells but the presence of somatic and non-spermatozoa germline cells is rare and associated to inflammatory processes of the reproductive tract (Michael Kleve-Feld, Personal Communication). Our group has always purified the porcine ejaculates with BoviPure^TM^ when characterizing the boar sperm transcriptome (5, 6, 8, 15) to be on the safe side since the potential impact of an even tiny presence of somatic cells on the semen transcriptome profile is unknown. The objective of this pilot study was to assess the changes on the porcine ejaculate's transcriptome after BoviPure^TM^ purification and discuss the potential underlying causes. To achieve this goal, we compared the transcriptomes of purified vs. paired non-purified ejaculates from four male pigs.

## Materials and Methods

### Sample Collection

Four fresh ejaculates showing good semen quality parameters (Supplementary Table 1) were obtained each from a different Pietrain boar from a commercial farm using the gloved hand method (16), diluted (1:2) immediately into the prepared fresh commercial extender and stored at 16°C. The ejaculates were privately owned for non-research purposes and the owners provided consent for their use for research. Specialized professionals at the farm collected the ejaculates following standard routine procedures and guidelines.

### Sample Processing and RNA Extraction

For each ejaculate, we collected two aliquots. One aliquot was purified (P) with the BoviPure^TM^ (Nidacon; Mölndal, Sweden) colloid centrifugation method. The other aliquot was not purified (NP). For the P samples, a maximum of 11 mL of ejaculate and 1 billion cells were placed over 3 mL of BoviPure^TM^ in 15 mL RNase-free tubes. The tubes were then centrifuged at 300 × g for 20 min at 20°C. After centrifugation, all the upper phases were removed. The cell pellet were transferred to a new 15 mL RNase-free tube with 10 mL RNase-free PBS and centrifuged at 1,500 × g for 10 min at 20°C. The supernatant was removed and the pellets were stored at −80°C in 1 mL Trizol® for further use for RNA extraction. A detailed description of the purification protocol with BoviPure^TM^ is provided by Gòdia et al. (5). For the NP aliquots, 1.5 mL of ejaculate was centrifuged at 14,000 g for 4 min at 20°C and the supernatant was removed. The pellet was then eluted in 1 mL of Trizol® and stored at −80°C until RNA extraction.

Total RNA was extracted from the eight samples as described in Gòdia et al. (5). Briefly, between 47 and 300 million cells were pre-lysed using a 5 mL sterile syringe with a 25 G needle for 5 and 2 min of vigorous vortex. After adding 200 μL of chloroform, the samples were incubated for 3 min at room temperature. Then, the samples were centrifuged at 12,000 × g for 15 min and the supernatants were transferred to new RNase-free tubes, in which 500 μL of isopropanol was added. These samples were then centrifuged at 12,000 × g for 10 min. The supernatants were removed carefully and the pellets were washed with 500 μL of 75% (v/v) ethanol solution. The samples were centrifuged at 13,000 × g for 5 min and dried out at room temperature for 10 min. The dried samples were resuspended in 30 μL of ultrapure water. All the centrifugations were performed at 4°C. All the RNA samples were subjected to DNase treatment with the Turbo DNA-free^TM^ kit (Thermo Fisher Scientific; CA, USA) following the manufacturer's instructions. The RNAs were quantified with Qubit^TM^ RNA HS Assay kit (Invitrogen; CA, USA).

In order to have an initial evaluation of the purity of the extracted RNA defined as RNA originating exclusively from sperm cells and devoid of DNA, for each sample, we used three qPCR assays that assess the abundance of the sperm specific *PRM1* RNA, the somatic-cell specific *PTPRC* RNA and the absence of genomic DNA (gDNA) using SYBR® Select Master Mix (Thermo Fisher Scientific; CA, USA) and done in triplicate as previously described (5). Total RNA abundance was quantified with Qubit RNA HS Assay kit (Invitrogen; CA, USA).

### RNA-Seq Library Preparation

Sequencing libraries were prepared with the SMARTer Stranded Total RNA-Seq Kit v2—Pico Input Mammalian (Takara Bio) using Dual Indexing strategy, starting with 10 ng of RNA quantified with the Qubit NRA HS Assay Kit (Thermo Fisher Scientific) and following the manufacturer's instructions. The final libraries were quality controlled on a Bioanalyzer (Agilent) using a High Sensitivity DNA Kit. The libraries were sequenced in an Illumina's HiSeq4000 system to generate 76 bp long paired-end reads following the manufacturer's protocol for dual indexing. Image analysis, base calling and quality scoring of the run were processed using the manufacturer's software Real Time Analysis (v 2.7.7) and followed by generation of FASTQ sequence files.

### RNA-Seq Mapping and Analysis

Total RNA-Seq reads were evaluated for quality control by FastQC software (v, 0.11.5) (https://www.bioinformatics.babraham.ac.uk). Low-quality reads (Phred –Q < 20 and read length < 25 bp) and sequencing adaptors were trimmed with Trimmomatic v.0.33 (17). Trimmed reads were aligned to the pig reference genome (Sscrofa 11.1) using HISAT2 v.2.1.0 (18) with the default parameters. Duplicate reads were removed with PicardTools v11.0.5 MarkDuplicates (http://picard.sourceforge.net). RNA levels of the genes annotated in the porcine genome (Ensembl v.101) were quantified as Fragments Per Kilobase of transcript per Million mapped reads (FPKM), with StringTie v.1.3.4 (19). The genes with FPKM ≤ 1 were excluded from further analysis. Differential gene abundance between P and NP was carried out by DESeq2 (20) and only these genes showing absolute log_2_ fold-change ≥ 1.5 and False Discovery Rate (FDR) ≤ 0.05 between P and NP were considered to be differentially abundant.

To determine whether the RNA-Seq quality metrics of the P and NP samples were significantly different, we used R to carry the Wilcoxon signed-rank test. The pairwise relationships between NP and the paired P transcriptomes were assessed by linear regression and with the Pearson correlation coefficient considering the purification treatment and the sample identifier. The hierarchical cluster dendrogram and the corresponding Selective Inference *p*-values were carried with the R package “pvclust” (21), and the Principal Component Analysis (PCA) and the correlation plot with the R package “ggplot2” (22). RNA transcript integrity (TIN) was calculated with RseQC v.2.6.4 (23) using the Ensembl v.101 pig annotation. TIN indicates the proportion of a gene that is covered by reads ranging from 0 (no coverage) to 100 (fully covered transcript). The TINs across paired samples was compared with the Wilcoxon signed-rank test.

To assess whether the differences in gene abundance identified between P and NP were really caused by the purification step and were not stochastic, we randomly shuffled the 8 samples into two groups of four samples each, 10 times and compared each time, the transcriptome profiles of the two groups with DESeq2 (20).

Gene Ontology (GO) enrichment analysis was performed with Cytoscape v.3.8.2 (24) plugin BiNGO v.3.0.4 using EBI porcine Gene Ontology Annotation Database (release: 2021-02-01) with default settings. Only the significant corrected *p*-values with FDR correction were considered.

### Evaluation of the Potential Tissue of Origin of the Differentially Abundant Genes Between Purified and Non-purified Samples

We carried an analysis to determine the potential tissue or cell type of origin of differentially abundant genes (DAG) between NP and P. We used a two-tailed Fisher's Exact Test to compare the proportion of genes with tissue-specific expression between the DAG and the non-DAG groups. We used an RNA-Seq dataset from an experiment carried by the Roslin Institute on 27 porcine tissues as part of the FAANG project (https://www.faang.org) with the NCBI's BioProject Accession Number PRJEB19386. The gene expression values (FPKM) of this dataset are available at the European Bioinformatics Institute ArrayExpress expression atlas (https://www.ebi.ac.uk/gxa/home) archive under file E-MTAB-5895. The catalog included two male and two female Duroc juveniles. All pigs were between four and six months old. The file provided tissue expression results from males and females separately. This dataset includes epididymis, penis, tonsil, amygdala, mesenteric lymph node, lung alveolus, liver, heart, adipose tissue, omentum, several central nervous system areas, skeletal muscle, spleen, several sections of the digestive tract, cortex of kidney, pituitary gland, and uterus. Tonsil was only analyzed in males. Obviously, the reproductive tissues were only represented by the one corresponding sex. We averaged the FPKM expression of the tissues analyzed in both males and females. We interrogated the genes with specific expression in epididymis as a male reproductive tissue and tonsil and lymph node as a tissue representing leukocytes which can be also be present in sperm. We also queried duodenum and skeletal muscle as two control tissues that are not expected to contribute cells to sperm.

We considered that a gene has a tissue-specific expression when its RNA FPKM in the target tissue was >20 and the FPKM in all the other tissues in the dataset was <5. Duodenum, tonsils and skeletal muscle did not show any tissue-specific gene. Thus, for these tissues we allowed up to three additional tissues with FPKM > 5. The genes identified following these conditions were considered as nearly tissue-specific. Then, we compared the abundance of these tissue-specific genes in the list of DAGs and the list of non-DAGs from our study.

We repeated this analysis using the GTEx Portal (dbGaP accession number phs000424.v8.ps), which contains RNA-Seq data from 54 human tissues including the prostate and testis male reproductive tissues as well as several representative of adipose tissue, artery, central nervous system, digestive tract, female reproductive organs, whole blood, EBV transformed lymphocytes, heart, kidney, skeletal muscle, skin, salivary gland, spleen, and thyroid. We used Ensembl BioMart (version 104) to identify the human orthologs of all the genes identified in our sperm (DAG and non-DAG) experiment. We queried the GTEx dataset using these human orthologous genes. We targeted prostate and testis as male reproductive tissues, EBV-transformed lymphocytes, and whole blood representing lymphocytes, which can be also found in sperm and liver, nerve tibial and mammary gland as control tissues which should not contain cell types that could be also found in sperm.

## Results and Discussion

### RNA Extraction and qPCR Results

RNA extractions showed very similar yields between the P and NP groups (Supplementary Table 2). Moreover, all the minus reverse transcription of the control samples showed no amplification of *PRM1* and *PTPRC* and the qPCR on the intergenic region was undetectable in all P and NP samples, thereby indicating no DNA contamination (Supplementary Table 2). The eight samples presented quantification cycles (Cq) ranging between 15.7 and 17.6 for the *PRM1* sperm-specific gene (Supplementary Table 2). In contrast, the Cq for *PTPRC* blood-specific gene for the P samples ranged between 35.6 and 37.2, and between 32.6 and 37.6 in NP (Supplementary Table 2). The ΔCq_*PTPRC*−*PRM*1_, calculated as the Cq for *PTPRC* minus the Cq for *PRM1* ranged between 18.1 and 21.0 in P, and 17 to 20.4 in NP, which means that, in average and as expected, the *PTPRC* assay had lower signal when compared to the *PRM1* assay in P than in NP (Supplementary Table 2). The NP aliquot of sample B2 showed more *PTPRC* signal (ΔCq_*PTPRC*−*PRM*1_) than any other of the eight samples thereby suggesting a degree of somatic cell presence in this sample.

### Sequencing Metrics

We sequenced an average of 67.6 million reads per sample. Of these, an average of 7.6 million reads were uniquely mapped to the porcine genome and used for downstream analysis (Supplementary Table 3). There were no significant differences between the sequencing metrics of P vs. NP samples (Supplementary Table 3).

### Description and General Comparison of the P and NP Transcriptomes

Using HISAT2, we identified an average of 7,519 protein coding genes with FPKM ≥ 1 and 1,870 genes with FPKM ≥ 10 (Supplementary Table 4). The genes displaying most abundant RNA levels included *PRM1, HSPB9, OAZ3, TSSK6*, and *TPPP2*, among others and were mostly related to sperm biology (Supplementary Table 4).

**Figure 1:**
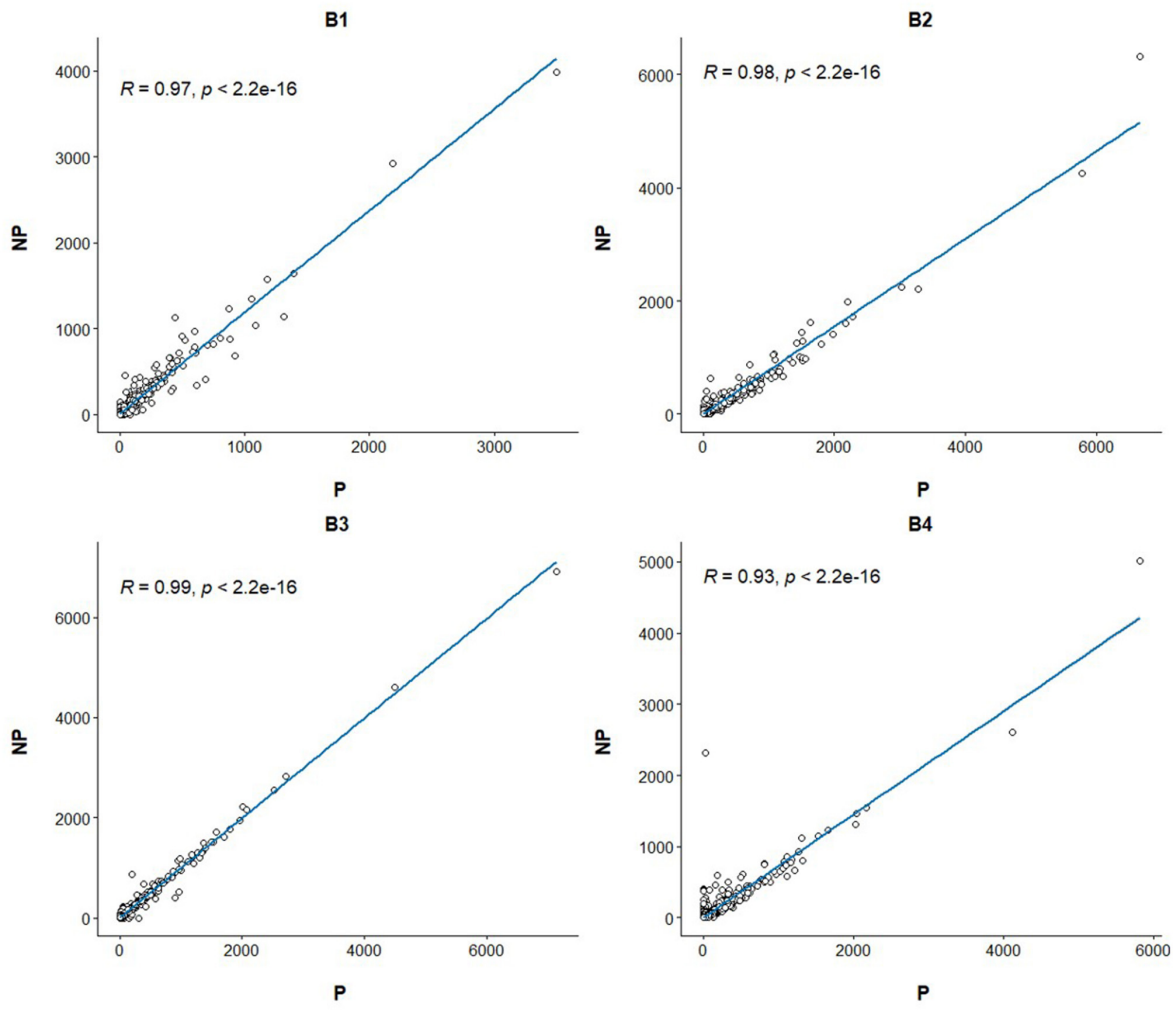
Linear regression and Pearson correlation of the transcriptome profiles between the purified and non-purified aliquots for the four ejaculates. The Pearson correlation and its *p*-value are annotated at the top left of each plot.

**Figure 2:**
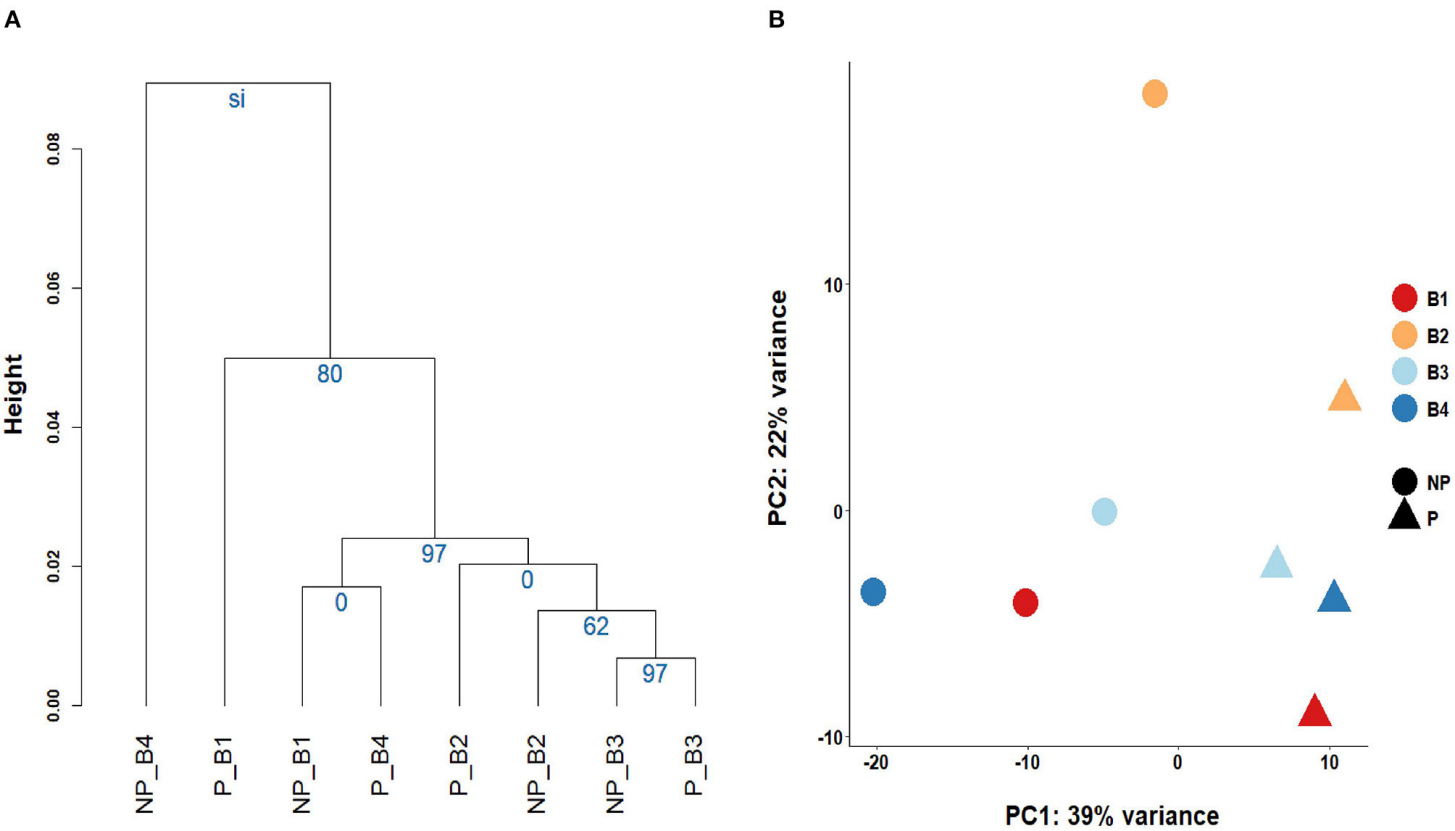
Grouping of the sperm transcriptomes of the eight samples. **(A)** Cluster analysis and the Selective Inference *p*-values. **(B)** Principal Component Analysis biplot.

Linear regression and correlation analysis based on the RNA levels of the 7,519 protein coding genes showed high correlations (between 0.93 in B4 and 0.99 in B3) between P and NP (Figure 1). Hierarchical clustering analysis of the 8 samples showed that the samples do not tend to group by the purification treatment but by their sample of origin (Figure 2A). Nevertheless, PCA seemed to separate NP and P into two different groups based on their principal component 1 (Figure 2B). Principal component 1 biggest contributors were *TTC38* followed by *PPARA, TRMU*, and *FAM118A*, which together explained 57.2% of this component (Supplementary Table 5). We searched the GTEx portal (dbGaP accession number phs000424.v8.ps) to determine the expression of these genes in human tissues. *TTC38* is mostly expressed in liver, small intestine, colon and whole blood and *PPARA* and *TRMU* have ubiquitous expression. On the contrary, *FAM118A* displays highest expression in testis, where spermatogenesis takes part, and prostate, which, at least in humans (25) and horse (26), contributes exosomes and can also provide epithelial cells to the human ejaculates (27). *TTC38*'s and *FAM118A*'s functions are not well known. *PPARA* is a transcription factor involved in energy metabolism and mitochondrial and peroxisomal function (28). *TRMU* is a mitochondrial tRNA modifying gene (29) thus related to protein synthesis. Sample B4 showed the largest disparity between NP and P of all the samples in all these analyses.

Contrarily to what happens in somatic cells, most sperm RNAs are fragmented. Thus, we also evaluated RNA fragmentation by measuring TIN, which ranges between 0 and 100 indicating from full fragmentation to full integrity of the transcript, and found similar (*p* = 0.37) TIN in the NP [mean ± standard deviation (SD) = 19.44 ± 16.04] and the P (mean ± SD = 17.89 ± 15.45) samples thereby indicating no major differences between both groups (Supplementary Table 6). This is probably due to the fact that the DAGs total RNAs represent a small proportion of the whole sperm transcriptome abundance (Supplementary Table 4).

### Differential RNA Abundance Analysis Between P and NP

We also carried differential abundance analysis to identify these genes with different RNA levels in P when compared to NP, a scenario that could be potentially caused by the removal of somatic cells, premature germline cells, immature, and morphologically abnormal spermatozoa or seminal plasma exosomes from the ejaculates. We used the 7,519 genes with FPKM ≥ 1 for the differential analysis using a paired sample design. Three hundred and seventy-two of the genes with FPKM ≥ 1 and 81 of the genes with FPKM ≥ 10 showed significant differential RNA abundance between P and NP (Supplementary Table 4) and were thus considered DAGs. To check whether the list of DAGs was stochastic or really owed to technical differences produced by the purification step, we randomly shuffled 10 times the eight samples into two four-sample groups and carried differential gene abundance analysis between both groups. These analyses showed between none and five DAGs in each comparison (Supplementary Table 7), thereby suggesting that the purification step has an impact on the RNA abundance of some genes. Most of the 372 DAGs showed a decreased RNA abundance in the P samples (Supplementary Table 4) which indicates that some cell types, organelles or vesicles were removed during the purification step. Only *TNNI3, CTXN1, SLC27A5, HSPA12B*, and ENSSSCG00000032730 presented increased RNA levels in P in at least three of the four samples (Supplementary Table 4). According to the GTEx portal (dbGaP accession number phs000424.v8.ps), *TNNI3*, a component of the striated muscle filaments, is exclusively expressed in the heart although one study linked it to prostate cancer (30). *CTXN1* is most highly expressed in the brain but it is also present at lower levels in several other tissues including testis, and its function is related to brain biology (31). *SLC27A5* is exclusively expressed in liver and is associated to lipid metabolism and bile synthesis (32). *HSPA12B* is highly abundant in spleen, lung, breast, and adipose tissues and has been related to heart injury (33) and lung cancer (34). Thus, no particular link with the cells expected to be found in the ejaculates could be drew for these genes. *TNNI3, SLC27A5, HSPA12B*, and ENSSSCG00000032730 presented low average abundance across the eight samples (FPKM <5) and even if significant, this data, should be considered with caution as it could be spurious. *CTXN1* displayed a much larger average abundance (FPKM = 85) and it is more likely to be real. Its increased levels in P indicate that a particular entity in the ejaculate—perhaps a particular sub-population of sperm cells—carrying this gene are more likely to be present after purification. However, as this is the only gene showing clear presence in the sperm and an increase in P, we cannot exclude the possibility that this was a spurious result. The 372 DAGs were enriched for GO terms related to translation and transcription and metabolic processes (Supplementary Table 8) and in fact, the list contained a large number of ribosomal proteins (e.g., *RPS3A, RPS27A*, and *RPL7*). The DAG group was clearly enriched over the non-DAG list for RPL and RPS ribosomal protein genes (Two-Tailed Fisher's Exact Test *p* <1.9E-53), which showed decreased levels in P when compared to NP. The majority of the DAGs showed an average abundance below 10 FPKM (Supplementary Table 4). Among the list of DAGs with average abundance above 10 FPKM, the top 10 that showed most significant differences included four ribosomal proteins and six genes with distinct functions (Table 1). These 10 genes are expressed in a wide catalog of tissues (GTEx portal; dbGaP accession number phs000424.v8.ps), but preferentially in EBV-transformed lymphocytes and most of them also in the female reproductive organs (Table 1). *PTMA* is related to cell proliferation (35), immunity (36), and chromatin remodeling (37) and it is also expressed in rat spermatocytes and spermatids but its functions in these cells are unknown (38). *NANS* is involved in the synthesis of sialic acid in multiple cell types and cellular processes (39) and within the catalog of human tissues, it is most highly expressed in the prostate but it is also present in other tissues including sperm. Sialic acid is a key component of the sperm glycocalyx assembled during sperm development, maturation and upon contact with the seminal fluid and is related to sperm. *TMSB4X* is linked to actin polymerization and cytoskeleton organization (40) and is most highly expressed in EBV transformed lymphocytes. *EEF1A1* delivers tRNA to the ribosome for translation elongation (41). One study carried RNA-Seq on chromatoid bodies isolated from testicles from GRTH knock-in mice with a sterile phenotype lacking elongated spermatids and spermatozoa (42). Chromatoid bodies are typically present in the cytoplasm of spermatocytes and round spermatids but are absent in elongating spermatids and spermatozoa and seem to be crucial for spermatogenesis. In this study, the authors identified an increased presence of genes related to transcript regulation, including *EEF1A1* in the mutant when compared to the wild type mice. *RACK1* acts as a scaffold assisting and modulating protein-protein interactions to recruit, assemble or regulate signaling molecules (43) and is a component of the 40S ribosomal subunit (44). The interaction between RACK1 and the AChE-R acetylcholinesterase splice variant in the spermatocytes of transgenic mice overexpressing *AChE-R* and displaying reduced sperm differentiation and sperm count showed that the interactions between AChE-R with RACK1 and enolase-α may be related to these sperm related phenotypes (45).

**Table 1:** Ten most differentially abundant genes with FPKM ≥ 10 between the purified and paired non-purified samples.

**Ensembl identifier**	**Gene name**	**FDR**	**Average FPKM**	**B1 P/NP**	**B2 P/NP**	**B3 P/NP**	**B4 P/NP**	**Average P/NP**	**Human tissues with highest expression according to the GTEx Portal**
ENSSSCG00000041806	–	5.74E-14	40	0.06	0.11	0.29	0.17	0.16	
ENSSSCG00000037274	Prothymosin Alpha (*PTMA*)	2.31E-12	32	0.08	0.31	0.14	0.07	0.15	Quite ubiquotous but mostly expressed in EBV-transformed lymphocytes. Low expression in testis.
ENSSSCG00000005373	N-Acetylneuraminate Synthase (*NANS*)	2.31E-12	16	0.03	0.23	0.30	0.10	0.16	Highest expression in the prostate and colon.
ENSSSCG00000009019	Ribosomal Protein S3A (*RPS3A*)	2.92E-12	13	0.10	0.26	0.17	0.06	0.15	Mostly expressed in the female reproductive organs and EBV-transformed lymphocytes.
ENSSSCG00000032111	Ribosomal Protein L7 *(RPL7*)	1.86E-11	15	0.09	0.47	0.14	0.14	0.21	Mostly expressed in the female reproductive organs and EBV-transformed lymphocytes.
ENSSSCG00000012119	Thymosin Beta 4 X-Linked (*TMSB4X*)	3.66E-11	16	0.09	0.16	0.12	0.20	0.14	Mostly expressed in EBV-transformed lymphocytes, spleen, lung, and whole blood.
ENSSSCG00000004489	Eukaryotic Translation Elongation Factor 1 Alpha 1 (*EEF1A1*)	4.45E-11	93	0.07	0.48	0.29	0.10	0.24	Mostly expressed in cultured fibroblasts, EBV-transformed lymphocytes, ovary, and uterus.
ENSSSCG00000033019	Ribosomal Protein L12 (*RPL12*)	5.8E-11	16	0.05	0.20	0.17	0.10	0.13	Most highly expressed in EBV-transformed lymphocytes, ovary, cultured fibroblasts, and cervix.
ENSSSCG00000029724	Receptor For Activated C Kinase 1 (*RACK1*)	6.6E-11	19	0.09	0.26	0.28	0.17	0.20	Most highly expressed in ovary, EBV-transformed lymphocytes, cultured fibroblasts, and cervix.
ENSSSCG00000031088	Ribosomal Protein S7 (*RPS7*)	7.0E-11	81	0.10	0.58	0.33	0.28	0.32	Most highly expressed in ovary, EBV-transformed lymphocytes and cultured fibroblasts.

*B1 P/NP, Ratio of the FPKM in the purified and the FPKM in the non-purified in B1; B2 P/NP, Ratio of the FPKM in purified and the FPKM in non-purified in B2; B3 P/NP, Ratio of the FPKM in purified and the FPKM in non-purified in B3; B4 P/NP, Ratio of the FPKM in purified and the FPKM in non-purified in B4; Average P/NP, Average of the ratio of the FPKM in purified and the FPKM in non-purified in the four samples*.

To get a glimpse of the tissue or cell type of origin of the DAGs group, we compared the proportion of the 372 DAGs with preferential expression in the porcine epididymis, a male reproductive organ known to contribute RNAs to the ejaculate (46), the tonsils and lymph node, that contain leukocytes, which could be also present in sperm. We also queried duodenum and skeletal muscle as control tissues that should not contain cell types also present in sperm. Three hundred and sixty-six DAG and 6,992 non-DAG genes were also present in the E-MTAB-5895 porcine gene expression catalog. Fifty-seven genes displayed specific expression in the epididymis, 5 and 13 of which were DAG and non-DAG, respectively (Table 2). Thus, 1.3% of the DAGs and 0.2% of the non-DAGs showed epididymis-specific expression. The two-tailed Fisher's Exact Test showed a clear difference (*p* = 0.001) between the abundance of epididymis specific genes in the DAG list when compared to the non-DAG group. Skeletal muscle and duodenum showed 48 and 5 tissue-specific genes, respectively. Five and one genes showed tissue-specific expression in the tonsils and lymph node, respectively, but none of these was present in our porcine sperm samples. For these three tissues we allowed up to three additional tissues with FPKM > 5. None of these four tissues showed a statistical difference between DAGs and non-DAGs (Table 2).

**Table 2:** Number of tissue-specific or nearly tissue-specific genes in the DAG and non-DAG groups and *p*-value of the comparison of the proportion of these genes in both groups.

**Tissue**	**Number of tissue-specific genes**	**Number of tissue-specific genes present in pig sperm**	**Number of nearly tissue-specific genes**	**Number of nearly tissue-specific genes present in pig sperm**	**Number in DAG**	**Number in non-DAG**	***P*-value**
Epididymis	57	18	N/A	N/A	5	13	0.001
Skeletal muscle	48	6	N/A	N/A	0	6	1
Duodenum	5	2	N/A	N/A	0	2	1
Lymph node	1	0	14	1	0	1	1
Tonsils	5	0	50	10	2	8	0.08
Testis	908	295	N/A	N/A	0	295	5.40E-07
Prostate	5	0	16	3	1	2	0.14
EBV transformed lymphocytes	13	0	149	40	0	40	0.26
Whole blood	7	0	105	6	0	6	1
Mammary	0	0	7	0	0	0	1
Liver	85	3	N/A	N/A	0	3	1
Nerve tibial	2	1	N/A	N/A	0	1	1

*Number in DAG, number of tissue-specific or nearly tissue-specific genes in the DAG group. Number in non-DAG, number of tissue-specific or nearly tissue-specific genes in the non-DAG group. P-value, P-value of the two-tailed Fisher's Exact Test comparing the proportion of tissue specific or nearly tissue-specific genes in the DAG vs. the non-DAG groups. N/A, non applicable*.

We carried the same analysis on the dataset of gene expression in 54 human tissues from the GTEx Portal (dbGaP accession number phs000424.v8.ps) querying testis and prostate as male reproductive organs, EBV transformed lymphocytes and whole blood as representatives of leukocytes and liver, nerve tibial and mammary gland as negative controls. Three hundred and twenty-seven and 6,304 DAGs and non-DAGs had a human ortholog present in the GTEx catalog (Table 2). Only testis, liver, and nerve tibial showed tissue-specific genes (Table 2). For prostate, EBV transformed lymphocytes, whole blood and mammary gland, we selected those genes which showed FPKM > 20 in the target tissue and FPKM > 5 in not more than three additional tissues. The list of testis-specific genes were the only ones that showed a statistically significant difference between DAGs and non-DAGs (*p* = 5.4E-07) but these genes were enriched in the non-DAG group (Table 2).

The genes *HBB, CDH1, PTPRC, KRT1, KRT10, CXCL8*, and *KLK3* previously used to determine the presence of leukocytes, epithelial cells, and prostate in human (47) and pig (5) showed no detectable levels of RNA in P and NP. Only *HBB* presented moderate abundance (FPKM = 36) in the NP sample from B4 (Supplementary Table 4). This data indicates that most NP samples are free of detectable levels of RNAs from somatic cell origin but that B4 may have contained a sufficient proportion of leukocytes to provide detectable levels of *HBB*. This is in line with the results on the cluster and PCA analyses which showed that the NP sample of B4 clustered and mapped apart from all the other samples and the fact that this sample showed the highest abundance for 266 of the 372 DAGs.

In light of these results, we wanted to explore whether the contribution of each sample to the list of DAGs was homogeneous or on the contrary, there was high variability between samples. We carried four differential abundance analyses between P and the paired NP samples but this time, removing each time, a different sample and thus carrying the comparison on only three ejaculates. The results demonstrated that the removal of sample B4 caused the most dramatic reduction of the number of DAGs when compared to the original differential abundance analysis carried on the four samples (Supplementary Table 9). Despite this outcome, the correlation between the NP and P transcriptomes of B4 was still high (*R*^2^ = 0.93) which shows that even in this sample, the BoviPure^TM^ purification did not have a dramatic impact of the overall transcriptome profile of this sample. Remarkably, the removal of B2 did not show any relevant change in the number of DAGs which, contrarily to what was indicated by the ΔCq_*PTPRC*−*PRM*1_, suggests no particular contamination of somatic cell RNAs. The semen quality phenotypes of the fresh ejaculate of B4 before purification showed lower ejaculate volume but all the other parameters (sperm cell concentration, sperm cell viability, percentage of spermatozoa with head, tail, or neck abnormalities, percentage of sperm cells with proximal or distal droplets and percentage of motile spermatozoa), did not deviate from the values observed in the other three samples (Supplementary Table 1). Therefore, we could not link the difference on the transcriptome of this sample to the semen quality phenotypes.

A somehow surprising finding was the clear enrichment of RPL and RPS ribosomal protein genes in the DAG group over the non-DAG list (Two-Tailed Fisher's Exact Test *p* < 1.9E-53), which showed decreased abundance in P when compared to NP. As intact ribosomal RNAs (rRNAs) are depleted in sperm to warrant translational silencing (48), we hypothesize that spermatozoa may also have reduced RNA levels of proteins forming the ribosomal sub-units. If true, this would indicate that the DAGs are contributed mostly by non-sperm entities.

This is also supported by the enriched abundance of human testis specific genes in the non-DAG list. We hypothesize that these non-DAG genes are specific to the later stages of spermatogenesis and they are present in the mature spermatozoa, which are not removed by the BoviPure^TM^ treatment. As a matter of fact, the list of 295 sperm-specific genes present in the non-DAG include important genes related to sperm biology and function such as *PRM1, PRM2, CATSPER1, CATSPERD, DAZL*, and several members of the *SPATA* and *SPACA* families among others (Supplementary Table 10).

As reviewed by Fedder in 1996, research in human semen has identified the presence of leukocytes, squamous epithelial cells, Sertoli cells, spermatogonia, spermatocytes, and spermatids (49). In addition, the presence of epithelial cells from the epididymis (50) and the prostate (27) have been also confirmed. In swine, sperm from artificial insemination studs rarely contain leukocytes but low levels of epithelial cells are frequently found (Michael Kleve-Feld, Personal Communication). We detected 57 pig epididymis-specific genes, 18 of which were present in our boar sperm samples. The DAG group was enriched for such genes, which indicates the removal of epididysomal cells or exosomes after the purification. On the other side, we did not find statistically significant differences in the proportion of human prostate-specific genes between the DAG and the non-DAG groups. This could be indicating that the prostate did not contribute a detectable level of epithelial cells or exosomes to the pig sperm but it could also be caused by the small number of human prostate's nearly specific genes, only 16, that we identified in the study, with resulted in only one and two genes present in the DAG and the non-DAG lists, respectively. Notwithstanding, the absence of detectable levels of prostate cells in sperm is also supported by the null presence of *KLK3* in all the NP and P samples.

The presence of epididymal material in sperm could be due to either epithelial cells or exosomes or a combination of both. Seminal exosomes have been found in the boar sperm (51). RNA-Seq data from human (52) and porcine (46) seminal exosomes showed that the RNA cargo of these vesicles is mostly made up of short RNAs but also include protein-coding RNAs. In pigs, RNA-Seq of the small RNA fraction of seminal exosomes from ejaculates displaying normal semen quality parameters showed that 25.3% of the sequencing reads corresponded to messenger RNAs (mRNAs). As the objective of this study was on microRNAs, the authors did not provide additional detail on the messenger RNAs present in the boar exosomes (46). Whether the BoviPure^TM^ purification removes the seminal vesicles is unknown. From the one side, BoviPure^TM^ separates the seminal fluid from the mature spermatozoa which should eliminate the exosomes present in the fluid. But on the other side, seminal exosomes also bind to the sperm's cell membrane and their removal after gradient-based protocols is uncertain. We don't know whether these exosomes unbind from the sperm during the purification or whether the sperm cells bound to exosomes have a distinct density that could remove them after purification. This also happens for the NP samples as they were subjected to a 14,000 g centrifugation for 4 minutes. Exosomes sedimentation often uses ultra-centrifugations of at least 120,000 g for 70 min. However, whether the sperm bound exosomes will sediment alongside with the sperm is unsure. In consequence, we hypothesize that the most likely largest contributor to the DAG list are epididysomal epithelial cells. A somatic cell has been estimated to contain in average between 10 and 30 picograms of RNA, in contrast with an average of 15 femtograms for a human spermatozoon. In addition, our own estimation suggests that the porcine sperm contains in average 1.6 femtograms per cell (5). Hence, the presence of an even tiny proportion of these epithelial cells in the sperm might be able to contribute detectable levels of RNA in the sample.

In order to further identify the origin of the RNAs impacted by BoviPure^TM^, future studies should be carried. To determine the contribution of abnormal or immature spermatozoa, the comparison should include an additional control consisting of an aliquot treated with somatic cell lysis buffer or sonication. The comparison between the three conditions should be still taken with caution as the SDS present in the lysis buffer could solubilise the sperm's membrane and remove its midpiece, which contains a large number of mitochondria thereby altering the resulting transcriptome (13). The contribution of the seminal exosomes could be further elucidated by purifying these vesicles by either ultra-centrifugation, or filtration by gradient based protocols and comparing the resulting transcriptome with that of the NP and P samples. This experiment should be accompanied by the exploration under the electron microscope of extracellular vesicles in the purified and non-purified samples. In any case, the results identified in swine cannot be translated to humans because the pig semen contains a much lower proportion of somatic cells than the men's ejaculate.

## Conclusion

Three of the four ejaculates had undetectable levels of somatic cell RNA markers before purification, thereby showing an agreement with the low levels of non-sperm cells typically found in samples from boar artificial insemination studs. We found strong indications that the purification with BoviPure^TM^ has a mild but noticeable impact on the RNA abundance of some genes and that this was originated by the removal of non-sperm cell entities with the most likely contribution of epididymal epithelial cells. The evaluation of the 10 most differentially abundant genes also indicated the removal of premature germline cells—even immature or morphologically abnormal spermatozoa—as well as leukocytes with a distinct RNA cargo. In light of these results, our group feels comfortable with the use of non-purified samples for the interrogation of the relationship between spermatozoon RNAs and semen quality traits. However, this should be decided by each research group in accordance to the experimental design and objective of their study.

## Data Availability Statement

The data presented in the study are deposited in the NCBI's short read archive repository, accession numbers SRR14117413, SRR14117412, SRR14117411, SRR14117410, SRR14117409, SRR14117408, SRR14117407, and SRR14117406.

## Ethics Statement

Ethical review and approval was not required for the animal study because this study was done on ejaculates that were privately owned by a commercial farm for non-research purposes. The owners provided consent for their use for research. Specialized professionals at the farm collected the ejaculates following standard routine procedures and guidelines.

## Author Contributions

AS and ACl conceived and designed the experiments. SB collected the samples. JR-G carried the phenotypic analysis. ACa carried the qPCR analyses. MG performed sperm purifications and RNA extractions. YL and MG made the bioinformatics and statistics analysis. YL, MG, and ACl analyzed the data. ACl, YL, JR-G, and MG wrote the manuscript. All authors discussed the data and read and approved the contents of the manuscript.

## Conflict of Interest

SB was employed by the company Group Gepork S.A. The authors declare that this study received funding from MINECO, AGAUR, Generalitat de Catalunya and MICINN. The funder was not involved in the study design, collection, analysis, interpretation of data, the writing of this article or the decision to submit it for publication. The remaining authors declare that the research was conducted in the absence of any commercial or financial relationships that could be construed as a potential conflict of interest.

## Supplementary Material

Supplementary Table 1 Phenotypic values for several semen quality parameters of the four ejaculates used in this study.

Supplementary Table 2 RNA extraction and qPCR data. P_B1, Purified sample of B1; NP_B1, Non-purified sample of B1; P_B2, Purified sample of B2; NP_B2, Non-purified sample of B2; P_B3, Purified sample of B3; NP_B3, Non-purified sample of B3; P_B4, Purified sample of B4; NP_B4, Non-purified sample of B4; P_average, Average value of the purified samples; NP_average, Average value of the non-purified samples.

Supplementary Table 3 Sequencing metrics. P_B1: Purified sample of B1; NP_B1: Non-purified sample of B1; P_B2: Purified sample of B2; NP_B2: Non-purified sample of B2; P_B3: Purified sample of B3; NP_B3: Non-purified sample of B3; P_B4: Purified sample of B4; NP_B4: Non-purified sample of B4.

Supplementary Table 4 Gene abundance across the eight samples for the 7,519 protein coding genes with average FPKM ≥ 1. DAG, Differentially abundant gene; FDR, False Discovery Rate; P_B1, Purified sample of B1; NP_B1, Non-purified sample of B1; P_B2, Purified sample of B2; NP_B2, Non-purified sample of B2; P_B3, Purified sample of B3; NP_B3, Non-purified sample of B3; P_B4, Purified sample of B4; NP_B4, Non-purified sample of B4.

Supplementary Table 5 Major gene contributors to principal component 1 of the Principal Component Analysis.

Supplementary Table 6 Transcript Integrity Number values across the eight samples. P_B1, Purified sample of B1; NP_B1, Non-purified sample of B1; P_B2, Purified sample of B2; NP_B2, Non-purified sample of B2; P_B3, Purified sample of B3; NP_B3, Non-purified sample of B3; P_B4, Purified sample of B4; NP_B4, Non-purified sample of B4.

Supplementary Table 7 Results of the 10 shuffles to organize the eight samples into 2 four-sample random groups. P_B1, Purified sample of B1; NP_B1, Non-purified sample of B1; P_B2, Purified sample of B2; NP_B2, Non-purified sample of B2; P_B3, Purified sample of B3; NP_B3, Non-purified sample of B3; P_B4, Purified sample of B4; NP_B4, Non-purified sample of B4. Random shuffles are identified as Random1, Random2, Random3, Random4, Random5, Random6, Random7, Random8, Random9, Random10. A and B denote the randomly generated groups in each shuffle.

Supplementary Table 8 Results of the Gene Ontology analysis of the 372 differentially abundant genes between the purified and the non-purified samples.

Supplementary Table 9 List of differentially abundant genes when including the four ejaculates and when removing the ejaculates B1, B2, B3, or B4. The second row shows the number of DAGs identified in that analyses. Down, gene showing significantly decreased RNAs levels in P when compared to NP. Up, gene showing significantly increased RNAs levels in P when compared to NP.

Supplementary Table 10 List of tissue-specific or nearly tissue-specific genes for pig epididymis, lymph node, tonsil, duodenum and skeletal muscle and for human prostate, testis, EBV transformed lymphocytes, whole blood, liver, and nerve tibial that are also present in the pig sperm. The last column shows whether that particular gene belonged to the DAG or the non-DAG group.

## Abbreviations

Cq: quantification cycle
DAG: differentially abundant gene
FPKM: fragments per kilobase of transcript per million mapped reads
GO: Gene Ontology
NP: non-purified sample
P: purified sample
PCA: Principal component analysis
TIN: transcript integrity number
tRNA: transfer RNA.
